# Civil society’s perception of forest ecosystem services. A case study in the Western Alps

**DOI:** 10.3389/fpsyg.2022.1000043

**Published:** 2022-09-29

**Authors:** Stefano Bruzzese, Simone Blanc, Valentina Maria Merlino, Stefano Massaglia, Filippo Brun

**Affiliations:** Department of Agricultural, Forest and Food Sciences (DISAFA), University of Turin, Turin, Italy

**Keywords:** forest ecosystem services, Best-Worst Scaling, latent class analysis, civil society, awareness, perception

## Abstract

Forest Ecosystem Services (FES) are widely recognised by the society nowadays. However, no study in the literature has analysed a ranking of FES after the pandemic. This paper investigated civil society’s perception and knowledge toward these services; in addition, the presence of attitudinal or behavioural patterns regarding individual’s preference, was assessed. A choice experiment was conducted using the Best-Worst Scaling (BWS) method on a sample of 479 individuals intercepted in the Argentera Valley, in the Western Italian Alps. Results, showed a strong interest in biodiversity, aesthetic landscape quality and psychophysical health and a lower interest in provisioning services. Based on the individual preferences, civil society was clustered into five groups for FES, named “Hedonistic,” “Individualist with cultural and health interests,” “Sensitive to regulatory and utilitarian functions,” “Climate change sensitive” and “Livelihood and hedonistic wellbeing.” In general, there was a growing appreciation by civil society for the intangible services offered by the forest, driven by modern lifestyles and an interest in learning more about the provided services. Based on these elements, we believe that similar research should be extended to other mountain contexts to validate the results or to find new insights, and that it is now necessary to study how to involve civil society in decision-making processes of forest planning and management at a local level.

## Introduction

Over the last 50 years or so, ecosystem services (ES) have gained strong recognition from civil society for their importance, not only for the environment, but also for human beings (Lin et al., 2021). Several organisations have attempted to study ES and classify them into specific categories, not least because of the different spatial relationship between their demand and supply (Costanza, 2008). These include the Millennium Ecosystem Assessment (MEA) (2005) project, which first formally defined ES as “the multiple benefits that ecosystems provide to humans”; the study on The Economics of Ecosystems and Biodiversity (TEEB) (2010), which presented them as “the direct and indirect contributions of ecosystems to human well-being”, adding a new category called “habitat services”; and the classification proposed by the Common International Classification of Ecosystem Services (CICES), later revised in 2012, which defined them as “the contributions that ecosystems make to human well-being” (Haines-Young and Potschin-Young, 2012). Thanks to its hierarchical structure, the latter classification allows the identification of different levels of ES detail, thus reducing the translation problems arising from different classification systems that were not always comparable (VanderWilde and Newell, 2021).

The MEA classification, which defines four categories of ES: “supporting,” “provisioning,” “regulating” and “cultural,” was used in this study, also following its wide recognition in the literature (Afonso et al., 2021; Chanza and Musakwa, 2021; Chettri et al., 2021; Kim and Son, 2021). Supporting services have a long-term effect and serve the formation of other services although they are the only ones that do not directly benefit humans; they include soil formation, nutrient cycling, and primary production (Sharafatmandrad and Khosravi Mashizi, 2021). Provisioning services are the material benefits that can be derived from the ecosystem, such as timber, drinking water, and fuel (Yoshimura et al., 2021). Regulating services derive from the management of ecosystem processes and include carbon storage, water regulation, and protection against natural hazards (Kim and Kwon, 2021). Finally, cultural services are the intangible benefits, such as the psychophysical health, the aesthetic beauty of a landscape, and the tourism-recreational activities (Santos Vieira et al., 2021).

Mountain and forest ecosystems play a key role, recognised both at the EU level with the new EU Forest Strategy 2030 (Aggestam and Giurca, 2021) – a flagship initiative of the European Green Deal – and internationally with the UN Sustainable Development Goals (Goal 15) (Marín et al., 2021; Rimal et al., 2021). This recognition can be attributed to the services offered, among which, the provision of drinking water (Piaggio and Siikamäki, 2021), CO2 storage (Blanc et al., 2019), protection against natural hazards (Scheidl et al., 2020), mental and physical wellbeing, and recreational tourism activities (Liu et al., 2021) are growing in importance.

However, these services, to contribute to human well-being, need to be identified, mapped, and assessed from an ecological perspective; furthermore, to make more robust public policy decisions it is crucial to also analyse the social interest of ES to identify lack of awareness, information asymmetry and issues arising from different stakeholders (Castro-Díaz et al., 2022). In this sense, several authors have defined a relational value, i.e., a value capable of including virtues, principles and preferences linked to human-nature interaction and capable of uniting social sciences with natural sciences of conservation, valorisation, and environmental sustainability (Arias-Arévalo et al., 2018; Chan et al., 2018; Himes and Muraca, 2018).

Previous studies attempted to identify the demand for Forest Ecosystem Services (FES) and the willingness of civil society to pay for some of these services (Soto et al., 2018; Jo et al., 2021). Others have tried to estimate their value (Accastello et al., 2019; Rijal et al., 2021) or to provide spatial-based tools capable of quantifying, mapping, and valuing FES (Khalfaoui et al., 2020) or assessing payments for such services (Grilli et al., 2020; Sacchelli et al., 2021). However, few researchers have attempted to ask civil society to identify a ranking of ES. This approach has been adopted: on a specific category, such as cultural services (Kabaya et al., 2019), using simple approaches such as Likert scales (Lin et al., 2021) or, on specific services offered by the forest (Soto et al., 2018; Beckmann-Wübbelt et al., 2021). studies concluded the data collection phase prior to the COVID-19 pandemic (Gouwakinnou et al., 2019; Yang et al., 2019).

Based on these premises and to fill some of these gaps in the literature, our study aims to answer the following questions:

• RQ1) How have civil society’s awareness and perception of FES changed in the post-COVID era?
• RQ2) Are there different patterns of civil society attitudes and behaviours regarding preferences for forest ecosystem services?

To do so, we designed a questionnaire, using the Best-Worst Scaling (BWS) method, which can detect individual preferences, following a choice-based approach. We applied this method in a local Italian mountain context in order to (i) identify a ranking of FES by civil society; (ii) define homogeneous groups of subjects according to their preferences toward to the selected different FES.

In recent years, mountain forests have undergone transformation and expansion in terms of occupied area (Malandra et al., 2019; Garbarino et al., 2020) as a result of several factors, including socio-economic changes, such as industrialisation, urbanisation, and the consequent lower intensification of agricultural land use in mountains (Bruzzese et al., 2020), and political-legal factors, such as the introduction of natural constraints and the establishment of parks, protected areas and reserves (Tattoni et al., 2021).

These transformations, in both the civil society lifestyle and in the supply of ecosystem services, may suggest a change in their demand (Schirpke et al., 2020). Indeed, the 20th century has shown a sharp increase in the supply of provisioning services at the expense of regulating and biodiversity ones (Pereira et al., 2020). In 2019, there was a reversal of this trend: regulatory services came first, and a growing interest in cultural services made them equal to provisioning ones (Acharya et al., 2019). Given these changes, we propose the following hypothesis:

H1) in recent years, partly due to the current pandemic emergency, cultural services provided by the forest have become the most demanded FES by civil society.

The rest of the document is structured as follows: Section “Materials and methods” describes the study area, the theoretical basis of the BWS method, and the questionnaire design adopted. Sections “Results” and “Discussion” report and comment on the results in the light of the classifications and BW scores obtained. The last section concludes with the limitations of this study and its possible developments.

## Materials and methods

### Case study

The study area was the Argentera Valley, located in the Western Italian Alps in Piedmont, on the border with France (44°54′42.4″N 6°53′49.7″E). The valley has an area surface of about 340 hectares, with a wide altitudinal range from a minimum of 1,540 m a.s.l. to a maximum of 3,303 m a.s.l. and is part of the Site of Community Importance (SCI) of the Natura 2000 network (code IT1110053). The area was chosen because it is a popular destination all year round for tourism and recreational purposes, and because we assumed the visitors are very environmental aware, given that access by car requires the purchase of a €5 ticket.

### Survey design and best-worst scaling

A choice experiment was conducted face-to-face in August 2021, using a structured paper questionnaire, developed to define the perceptions and attitudes of a sample of subjects toward ecosystem services (Supplementary material A).

Interviews were conducted using the questionnaire administered throughout the day (from 9am to 5pm) considering the whole week (Monday to Sunday) to randomly intercept a sample as heterogeneous as possible. The eligibility criterion of the respondents was for over 17-year-olds. The questionnaire was structured in two sections: the first one dedicated to the socio-demographic characteristics of the individuals, a second part implemented a defined number of BWS questions. The BWS methodology was chosen because it allows defining the degree of preference (through a numerical index) toward a single item describing a product, an environment, a topic, etc., starting from a set of defined attributes/descriptors. This multivariate and quantitative method is based on pairwise comparisons (Shuibul Qarnain et al., 2021), offering several advantages if compared to other methods used for indirect assessment of individual preferences (Finn and Louviere, 1992; Marley and Louviere, 2005; Louviere et al., 2015). During the interviews, respondents were asked to choose the best and worst attributes to describe ecosystem services for each of several subsets (BWS questions) containing the previously selected FES characteristics in different combinations. This procedural approach allows us to overcome the limitations of ranging and ranking that imply a high cognitive effort of the respondent, thus reducing the efficiency of the survey (Marley and Louviere, 2005). The adopted BWS design was developed using the Sawtooth MaxDiff Designer software (SSI-version 8.4.6, Orem, UT, USA^1^) (Orme, 2009), following the standard balanced incomplete block design (BIBD) (Mori and Tsuge, 2017) with the following characteristics: starting from a set of n attributes, r choice sets (best-worst question) are provided, each containing t attributes (constant condition n > t). Therefore, each attribute appears s times in the experimental design and each pair of items appears α times [α = *s* × (*t*–1) / (*n*–1)] (Crouch and Louviere, 2007; Liu et al., 2018). During the interviews, respondents repetitively select the maximum difference couple of attributes (for each best-worst question). In the presented research, *n* = 12 attributes were selected (Table 1), organised in the questionnaire into *r* = 9 choice sets, each containing *t* = 4 attributes, and each attribute appeared *s* = 3 times in the experimental design. To further increase the combinations of attribute choices, four different versions of the questionnaire were developed in each of which the order of the attributes within the BWS questions changed.

**TABLE 1:** Attributes list and description.

Label	Description
Biodiversity	Plant and animal habitats
Aesthetic quality of the landscape	Beauty of the landscape
Psychophysical health	Reduces stress and strengthens the immune system
Protection against natural hazards	Avalanches, rockfalls and landslides
Recreational tourism	Hiking, mountain biking and camping
Disaster reduction	Flooding
Climate change mitigation	Carbon storage
Drinking water	Drinking water at the home tap
Raw materials	Construction timber, carpentry, and handicrafts
Food	Mushrooms, small fruits, fish, and game
Fuel	Firewood, pellets, and wood chips
Spiritual and religious	Pilgrimage and religious retreats

The analysis of the answers produced an average preference index (ARS) for the individual elements, which it was then used to rank the sample’s preferences toward the selected ecosystem services (Umberger et al., 2010). Specifically, the formula applied to calculate the ARS for a single attribute (i) is as follows:


$$A\,R\,S\,i=\frac{C\,O\,U\,N\,T\,b\,e\,s\,t-C\,O\,U\,N\,T\,w\,o\,r\,s\,t}{s*n}$$

where:

• COUNTbest represents the number of times the individual attribute was chosen as BEST (best);
• COUNTworst represents the number of times the single attribute was chosen as WORST (worst);
• s is the number of times the single attribute appears in the experimental design (s = 3);
• n is the sample size.

These preference scores (which measure the importance of each individual item) can be positive or negative, and their sum is always equal to zero. The standard deviation was used as a crude indicator of variability for defining the preferences of the whole sample.

### Latent class analysis

The relative scores (Rescaled Score – RS) (whose sum, considering all 12 attributes, is equal to 100) were used as dependent variables in Latent Class Analysis (LCA), in order to obtain homogeneous groups of subjects based on the individuals’ preferences (Casini et al., 2009; Massaglia et al., 2019). The use of RS in cluster analysis allows for comparing and better interpreting the differences between the obtained groups (Cohen, 2009). The theoretical properties of LCA are explained in Umberger et al. (2010). In general, starting from the characteristic of LCA which, contrary to other segmentation techniques, does not allow to knowing the number and the size of clusters obtained *a priori* and providing several usable solutions, following the suggestions of Dekhili et al. (2011), we selected the lowest values of the Log-Likelihood (LL) and the related Bayesian Information Criterion (BIC) for each model, choosing the best five-cluster model. The HSD ANOVA was conducted in SPSS 28.0 for Windows, using Tukey’s test to define significant differences in preferences among the five clusters (Tabacco et al., 2021).

## Results

### Sociodemographic characteristics

Details of socio-demographic variables of the 479 respondents are reported in Table 2. The selected sample is gender-balanced and the average age was 44.3 years. About two-thirds of the visitors were families with children, with a medium to a high level of education. Moreover, about 75% of the respondents visited the site less than 5 times a year and 85% of them came from urban or suburban areas.

**TABLE 2:** Sample characteristics.

Category	Item	Frequency	Percent
		**[n.]**	**[%]**
Gender	Female	234	48.9%
	Male	245	51.1%
Age groups	18–30	108	22.5%
	31–40	87	18.2%
	41–50	100	20.9%
	51–60	93	19.4%
	>60	91	19.0%
Family composition	1 (single)	28	5.8%
	2 (couple)	126	26.3%
	3	107	22.3%
	4	172	35.9%
	>4	42	8.8%
	n/a	4	0.8%
Educational level	Primary school	1	0.2%
	Lower secondary school	58	12.1%
	Upper secondary school	211	44.1%
	Master’s degree	209	43.6%
Site frequency (no. of visits/year)	1	206	43.0%
	2–5	151	31.5%
	6–10	43	9.0%
	11–20	21	4.4%
	>20	56	11.7%
	n/a	2	0.4%
Residence area	Urban area (City)	238	49.7%
	Small towns or suburbs	168	35.1%
	Rural area	59	12.3%
	n/a	14	2.9%

### Best-worst scores

**FIGURE 1:**
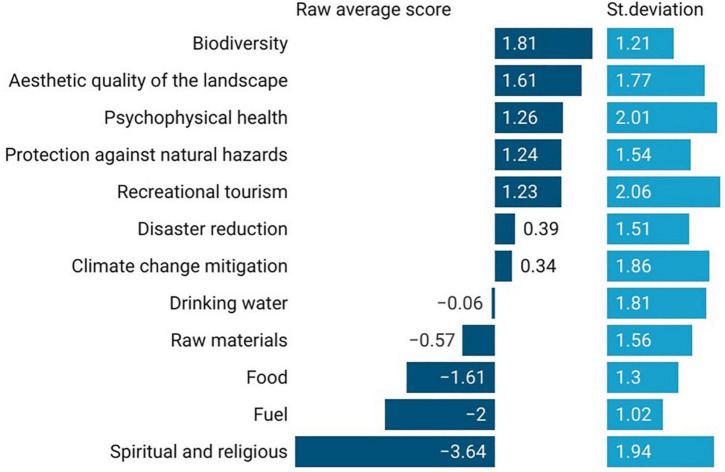
Raw average score for each FES attribute.

The Raw Average Score (RAS) of each attribute identified the average level of preference for the FES expressed by the respondents (Figure 1).

The first three preferred FES resulted from the respondents’ answers were biodiversity (with the highest average RAS of 1.81), aesthetic quality of the landscape (RAS = 1.61), and psychophysical health (RAS = 1.26). A general interest in livelihood, cultural, and well-being attributes was therefore expressed.

The least important attribute, on the other hand, was spiritual and religious (RAS = –3.64), in fact the explored case study area has never had a spiritual connotation and therefore visitors confirmed they did not access the area for such reasons. The others two attributes that had the lowest raw scores were: fuel (RAS = –2.00) and food (RAS = –1.61), thus highlighting how the needs of civil society have changed. While in the past these two attributes were essential to the lives of mountain people, they are now not perceived by users, who highlighted interests and needs related to contemporary life and linked to intangible services.

### Clustering results

The Latent Class analysis was performed considering the entire sample of respondents (*n* = 479) allowing the identification of 5 different groups of individuals (Table 3).

**TABLE 3:** Latent class analysis results showing the rescaled scores (relative preference index) for each FES attribute, resulting in the obtained 5 clusters.

	Average raw score				

Cluster name	Hedonistic	Individualist with cultural and health interests	Sensitive to regulatory and utilitarian functions	Climate change sensitive	Livelihood and hedonistic wellbeing
Cluster size	25.8%	22.4%	19.8%	18.0%	14.1%
**Attribute**					
Food	1.636 a	2.055 a, b	2.773 b	4.323 c	7.092 d
Drinking water	1.564 a	9.169 b	7.680 b	9.907 b, c	12.831 d
Raw materials	4.645 b	8.071 d	1.146 a	5.780 b, c	9.628 c
Fuel	1.179 a	1.656 b	2.923 c	2.396 c	4.435 d
Climate change mitigation	6.663 b	2.914 a	11.900 c	16.758 d	5.576 b
Disaster reduction	7.982 b	2.266 a	14.880 d	10.836 c	10.231 b
Protection against natural hazards	11.693 b	4.760 a	18.824 d	13.482 c	11.203b
Biodiversity	15.158 c	11.560 b	16.126 c	15.599 c	9.437 a
Aesthetic quality of the landscape	17.684 c	18.315 c	6.387 a	6.896 a	12.547 b
Recreational tourism	16.148 c	17.938 c	10.091 b	1.697 a	12.322 b
Spiritual and religious	0.530 a	1.761 b	0.768 a	0.744 a	1.996 b
Psychophysical health	15.117 d	19.535 e	6.501 b	11.583 c	2.703 a

a–d: preference averages (rescaled scores) within a row with different superscripts differ (*P* < 0.05) for Tukey *post-hoc* test.

The first cluster, called “Hedonistic,” identified respondents who used natural resources mainly for recreational tourism purposes, enjoying the aesthetic quality of the landscape, the beauty linked to biodiversity, and with the aim of improving their mental and physical health. Compared it to the next group, which is similar in terms of its positive assessment of the aesthetic quality of the landscape and recreational tourism, this group also stood out for rationally assessable aspects such as biodiversity and protection against natural hazards.

The second cluster, “Individualist with cultural and health interests,” identified respondents who used forests and mountains in their free time with tourist activities (walking, mountain-biking, hiking), for personal purposes linked to emphasising the pleasure of enjoying the beauty of the landscape to achieve psychophysical well-being, reducing stress, and strengthening the immune system.

The group “Sensitive to regulatory and utilitarian functions” was represented by respondents attentive to the regulatory aspects provided by ecosystems, both on a local scale (avalanches, rock falls, landslides) and, therefore, with reference to the active use of the territory, and on a basin and regional scale, considering the mitigation effect that the forest can guarantee with respect to calamitous events, also highlighting interest in aspects related to biodiversity.

The “Climate change sensitive” cluster identified users who were attentive to ongoing climate change and express a general interest in the capacity of natural ecosystems to actively contribute to mitigating the effects of global change, as well as guaranteeing protection from natural hazards and maintaining biodiversity.

Finally, the “Livelihood and hedonistic wellbeing” cluster identified respondents who placed the forest as an ecosystem at the service of humans, with the function of supplying drinking water and raw materials (such as timber for construction, carpentry, or objects), for its aesthetic qualities linked to the landscape and for the possibility of recreational activities.

In general, two attributes were common to at least three clusters in terms of the level of importance: Biodiversity and Aesthetic quality of the landscape. Aspects that together identified the strategic centrality of the examined area for tourism activities.

On the other hand, the five clusters agreed on defining Food, Fuel, Spiritual and Religious as not particularly important. This revealed the evolution of the concept of the resource itself and the abandonment of the traditional functions of supplying materials and food, and the absence of spiritual links with the natural resource examined.

## Discussion

Several authors highlighted the importance of the role attributed to FES for the environment, society, and economy (Acharya et al., 2019; Bussola et al., 2021; Kramer et al., 2022). In a review conducted by Nummelin et al. (2021) on the topics and trends of international forestry scientific research in the period 2000–2019, an increasing interest in such services emerged.

The recognition of FES by civil society led, on the one hand, for forest owners and managers to deal with an increase in their demand, as reported by Müller et al. (2020); on the other hand, as highlighted by Bonsu et al. (2017) in the creation of bottom-up initiatives that gave space for the population to participate in decision-making processes of forest planning and management.

In this context, it is therefore important to identify civil society’s perception of and interest in the FES to optimise the matching of supply and demand and to provide more robust information for decision-making. Based on these considerations, two key results emerge from our analyses:

1. Today, society’s awareness and perception of the FES has changed, accelerated by the current pandemic emergency. Our study reported biodiversity, aesthetic quality of the landscape, and psychophysical health as the most preferred services by civil society, while food, fuel, and spiritual and religious activities as the least. These results partially confirmed our hypothesis and what Acharya et al. (2019) stated in their study on the perception and prioritisation of ecosystem services by users and local stakeholders in a mountain context. Indeed, except for regulating services, which were of primary importance in both studies, cultural services received more recognition than provisioning services in our case. This could be attributable to the socio-demographic characteristics of our sample or, to the effects induced by the pandemic, which promoted greater frequentation of forests and natural environments, especially for recreational purposes or psychophysical well-being, as confirmed by several authors (Bamwesigye et al., 2021; Hansen et al., 2022; Jarský et al., 2022; Vos et al., 2022).

Conversely, the fact that food has been perceived as less important among ecosystem services, may be due to the respondents’ lack of knowledge about edible forest products (such as blueberries, mushrooms, and game), hence the need for awareness-raising campaigns, as reported by Gouwakinnou et al. (2019). The low interest in fuel is to be found in the socio-demographics of the sample, as most respondents come from urban backgrounds and probably do not use woody biomass for energy purposes. Finally, the little attention paid to religious and spiritual activities can probably be attributed to the specificity and distinctiveness of the service.

Before the pandemic emergency, one of few studies conducted in Italy on social perception was the one by Pastorella et al. (2016), but it is related to forest functions and not to ecosystem services, which are rather different concepts (Brun, 2002). Therefore, these studies referred to the capacity of the forest to provide goods and services (De Groot, 1992), rather than to the benefit produced by them for humans (Farber et al., 2002). In any case, the results seemed consistent with those obtained from our analyses, since the primary importance of biodiversity emerged, followed by that of the aesthetic quality of the landscape. This may indicate, on the one hand, that perceived importance is influenced by ecological knowledge and by the socio-economic and cultural context of a place, and on the other, that there was a matching between what stakeholders consider important from the forest and what civil society wants.

Conversely, at the international level, provisioning services continue to be of key importance. Lhoest et al. (2019) investigated the perception of forest ecosystem services by local communities in Cameroon. In a sample of 225 respondents, the primary interest was shown in provisioning services (93.3% of respondents), followed by cultural services (68%), and regulating services (16%). Zhang et al. (2019), conducted a study in China on a sample of 386 respondents, which showed a keen interest in provisioning and regulating services. Tauro et al. (2018), conducted a study in Mexico on a small sample of 27 livestock farmers, which showed that the most important ecosystem services include provisioning services (50%) and that the rest are given by a combination of supporting, regulating and cultural services. Finally, Haida et al. (2016) conducted a study on a sample of 53 decision-makers in mountain areas of Austria and Italy, which showed that most of them ranked provisioning services as the most important, followed by regulating and supporting services.

It seems logical to assume that these different perceptions are attributed to the different socio-cultural contexts, as stated by Caballero-Serrano et al. (2017). Indeed, local customs, dietary habits, proximity to the forest, dependence on the forest for work and income are all factors that contribute to influencing respondents’ preferences and should be kept in mind when interpreting the results of the study.

2. Civil society was classified into five groups in terms of FES preference. The “Hedonistic” group, which found pleasure in the sight of a beautiful landscape and in conducting recreational activities in the forest that also influence psychophysical health. The fact that this group emerged as the main one from our analyses may underline the statement already made by Koprowicz et al. (2022) in their study to determine the attitudes of Poles toward the forest during the COVID-19 pandemic. What emerged from a sample of 1025 participants was a visible societal need for forestry activities, which accrued particularly during the pandemic.

The second group, defined as “Individualists with cultural and health interests,” highlighted a marked interest in the wellbeing of the individual resulting from conducting activities in the forest. Several authors in the literature, in fact, highlighted the multiple physical and psychological benefits derived from forest recreation, such as stress reduction, immune system strengthening, and pressure reduction (Bielinis et al., 2019; Kotera et al., 2022; Muro et al., 2022; Roviello and Roviello, 2022).

The “Sensitive to regulatory and utilitarian functions” group placed the main emphasis on the safety and liveability of a place, therefore, presenting a greater interest in the protective role that the forest has against gravitational natural hazards and disasters, and in biodiversity as a principal element for the stability of an ecosystem. In a study conducted on the protective role of a forest stand against rockfalls, Lingua et al. (2020) pointed out that the protection offered by the forest in the mountains has always been considered prominent. Scheidl et al. (2020) also stressed the importance of mountain forests in reducing the risk of rockfalls over large areas and long periods of time.

The “Climate change sensitive” group was more concerned with an interest of global importance, which is the mitigation of the climate crisis. This group expressed an altruistic and legacy function, which can be partly traced back to the views commonly referred to as the “Greta generation” (Magnenat, 2021; Prakoso et al., 2021; Sabherwal et al., 2021).

The last group, defined as “Livelihood and hedonistic wellbeing,” emphasised cultural services, but also recognised the role of the forest in the provision of products as well as services and specifically drinking water. The fact that provisioning services were considered less interesting can be attributed to two causes. The first cause is due to the socio-demographic characteristics of the sample, as most of the subjects were users and not mountain dwellers and were probably not aware of the role of supply provided by a forest. The second cause may be attributable to the current trend regarding the FES, which as reported by Acharya et al. (2019), is more focused on regulating and cultural services than on provisioning and supporting services.

## Conclusion

This study sought to understand civil society’s perception and relative preference for forest ecosystem services in the post-COVID period. To our knowledge, this is the only post-pandemic study to have identified a ranking of FES belonging to the various categories defined by the MEA, using a choice-based approach. Our results highlight a strong interest of civil society in the component of biodiversity and cultural services, such as landscape aesthetic quality and psychophysical health, and based on individual preferences, identify different homogeneous groups of attitudes and behaviours toward FES. This is a small but significant step toward a better understanding of the forest-society relationships that underpin good policy and good governance on the part of decision-makers.

### Limitations and future research

There are two potential limitations to this study. The first concerns the characteristics of the sample, the results we have obtained probably reflect the preferences of a civil society that frequents the mountains as tourists and does not live there permanently. This may be one of the main reasons why provisioning and regulating services related to the safety and liveability of a place were not considered so important. Further research must be conducted in this respect, analysing several samples at a time, and assessing the differences between them. The second limitation concerns the choice of ecosystem services to be assessed in the questionnaire, which is based on only some of those proposed by the MEA classification. The reason for this choice is twofold: on the one hand, those most recognisable to civil society were selected; on the other hand, as the methodology is based on a process of choosing between several alternatives, there was a risk of spending too much time filling in the questionnaire.

In conclusion, our study can contribute to improving the participatory and shared decision-making process in forest planning and management, which considers the multiple interests deriving from the different components of society (authorities, stakeholders, and citizens) and are indispensable in resource decisions. Further research is required, however, to understand how and in what way to better involve civil society during the decision-making process.

## Data availability statement

The original contributions presented in this study are included in the article/Supplementary material, further inquiries can be directed to the corresponding author.

## Author contributions

SBr and SBl: conceptualization. SBr, SBl, and VM: methodology. SBr and VM: formal analysis. SBr, SBl, VM, SM, and FB: writing – original draft preparation and writing – review and editing. SM: supervision. All authors contributed to the manuscript revision, read, and approved the submitted version.

## Footnotes

1
http://www.sawtoothsoftware.com/

## References

[B1] Accastello C. Bianchi E. Blanc S. Brun F. (2019). ASFORESEE: A harmonized model for economic evaluation of forest protection against rockfall. *Forests* 10:578. 10.3390/f10070578

[B2] Acharya R. P. Maraseni T. Cockfield G. (2019). Global trend of forest ecosystem services valuation – An analysis of publications. *Ecosyst. Serv.* 39:100979. 10.1016/j.ecoser.2019.100979

[B3] Afonso F. Félix P. M. Chainho P. Heumüller J. A. de Lima R. F. Ribeiro F. (2021). Assessing ecosystem services in mangroves: Insights from São Tomé Island (Central Africa). *Front. Environ. Sci.* 9:501673. 10.3389/fenvs.2021.501673

[B4] Aggestam F. Giurca A. (2021). The art of the “green” deal: Policy pathways for the EU Forest Strategy. *Forest Policy. Econ.* 128:102456. 10.1016/j.forpol.2021.102456

[B5] Arias-Arévalo P. Gómez-Baggethun E. Martín-López B. Pérez-Rincón M. (2018). Widening the evaluative space for ecosystem services: A taxonomy of plural values and valuation methods. *Environ. values* 27 29–53. 10.3197/096327118X15144698637513

[B6] Bamwesigye D. Fialová J. Kupec P. Łukaszkiewicz J. Fortuna-Antoszkiewicz B. (2021). Forest recreational services in the face of COVID-19 pandemic stress. *Land* 10:1347. 10.3390/land10121347

[B7] Beckmann-Wübbelt A. Fricke A. Sebesvari Z. Yakouchenkova I. A. Fröhlich K. Saha S. (2021). High public appreciation for the cultural ecosystem services of urban and peri-urban forests during the COVID-19 pandemic. *Sustain. Cities Soc.* 74:103240. 10.1016/j.scs.2021.103240

[B8] Bielinis E. Bielinis L. Krupiñska-Szeluga S. Łukowski A. Takayama N. (2019). The effects of a short forest recreation program on physiological and psychological relaxation in young polish adults. *Forests* 10:34. 10.3390/f10010034

[B9] Blanc S. Accastello C. Bianchi E. Lingua F. Vacchiano G. Mosso A. (2019). An integrated approach to assess carbon credit from improved forest management. *Null* 38 31–45. 10.1080/10549811.2018.1494002

[B10] Bonsu N. O. Dhubháin ÁN. O’Connor D. (2017). Evaluating the use of an integrated forest land-use planning approach in addressing forest ecosystem services conflicting demands: Experience within an Irish forest landscape. *Futures* 86 1–17. 10.1016/j.futures.2016.08.004

[B11] Brun F. (2002). Multifunctionality of mountain forests and economic evaluation. *Forest Policy Econ.* 4 101–112. 10.1016/S1389-9341(02)00010-2

[B12] Bruzzese S. Blanc S. Brun F. (2020). Strategies for the valorisation of chestnut resources in Italian mountainous areas from a sustainable development perspective. *Resources* 9:60. 10.3390/resources9050060

[B13] Bussola F. Falco E. Aukes E. Stegmaier P. Sorge S. Ciolli M. (2021). Piloting a more inclusive governance innovation strategy for forest ecosystem services management in Primiero, Italy. *Ecosyst. Serv.* 52:101380. 10.1016/j.ecoser.2021.101380

[B14] Caballero-Serrano V. Alday J. G. Amigo J. Caballero D. Carrasco J. C. McLaren B. (2017). Social perceptions of biodiversity and ecosystem services in the ecuadorian amazon. *Hum. Ecol.* 45 475–486. 10.1007/s10745-017-9921-6

[B15] Casini L. Corsi A. M. Goodman S. (2009). Consumer preferences of wine in Italy applying best-worst scaling. *Int. J. Wine Bus. Res.* 21 64–78. 10.1108/17511060910948044

[B16] Castro-Díaz R. Delgado L. E. Langle-Flores A. Perevochtchikova M. Marín V. H. (2022). A systematic review of social participation in ecosystem services studies in Latin America from a transdisciplinary perspective, 1996–2020. *Sci. Total Environ.* 828:154523. 10.1016/j.scitotenv.2022.154523 35292319

[B17] Chan K. M. Gould R. K. Pascual U. (2018). Editorial overview: Relational values: What are they, and what’s the fuss about? *Curr. Opin. Environ. Sustain.* 35 A1–A7. 10.1016/j.cosust.2018.11.003

[B18] Chanza N. Musakwa W. (2021). Indigenous practices of ecosystem management in a changing climate: Prospects for ecosystem-based adaptation. *Environ. Sci. Policy* 126 142–151. 10.1016/j.envsci.2021.10.005

[B19] Chettri N. Aryal K. Thapa S. Uddin K. Kandel P. Karki S. (2021). Contribution of ecosystem services to rural livelihoods in a changing landscape: A case study from the Eastern Himalaya. *Land Use Policy* 109:105643. 10.1016/j.landusepol.2021.105643

[B20] Cohen E. (2009). Applying best-worst scaling to wine marketing. *Int. J. Wine Bus. Res.* 21 8–23. 10.1108/17511060910948008

[B21] Costanza R. (2008). Ecosystem services: Multiple classification systems are needed. *Biol. Conserv.* 141 350–352. 10.1016/j.biocon.2007.12.020

[B22] Crouch G. I. Louviere J. J. (2007). *International Convention Site Selection: A further analysis of factor importance using best-worst scaling.* Queensland: CRC for Sustainable Tourism.

[B23] De Groot R. S. (1992). *Functions of nature: Evaluation of nature in environmental planning, management and decision making.* Groningen: Wolters-Noordhoff BV.

[B24] Dekhili S. Sirieix L. Cohen E. (2011). How consumers choose olive oil: The importance of origin cues. *Food Qual. Prefer.* 22 757–762. 10.1016/j.foodqual.2011.06.005

[B25] Farber S. C. Costanza R. Wilson M. A. (2002). Economic and ecological concepts for valuing ecosystem services. *Ecol. Econ.* 41 375–392. 10.1016/S0921-8009(02)00088-5

[B26] Finn A. Louviere J. J. (1992). Determining the appropriate response to evidence of public concern: The case of food safety. *J. Public Policy. Market.* 11 12–25. 10.1016/S0140-6736(16)00619-X 27021149 PMC5042332

[B27] Garbarino M. Morresi D. Urbinati C. Malandra F. Motta R. Sibona E. M. (2020). Contrasting land use legacy effects on forest landscape dynamics in the Italian Alps and the Apennines. *Landsc. Ecol.* 35 2679–2694. 10.1007/s10980-020-01013-9

[B28] Gouwakinnou G. N. Biaou S. Vodouhe F. G. Tovihessi M. S. Awessou B. K. Biaou H. S. S. (2019). Local perceptions and factors determining ecosystem services identification around two forest reserves in Northern Benin. *J. Ethnobiol. Ethnomed.* 15:61. 10.1186/s13002-019-0343-y 31796089 PMC6889549

[B29] Grilli G. Fratini R. Marone E. Sacchelli S. (2020). A spatial-based tool for the analysis of payments for forest ecosystem services related to hydrogeological protection. *Forest Policy Econ.* 111:102039. 10.1016/j.forpol.2019.102039

[B30] Haida C. Rüdisser J. Tappeiner U. (2016). Ecosystem services in mountain regions: Experts’ perceptions and research intensity. *Reg. Environ. Change* 16 1989–2004. 10.1007/s10113-015-0759-4

[B31] Haines-Young R. Potschin-Young M. (2012). *CICES version 4: Response to consultation. Centre for environmental management.* Nottingham: University of Nottingham.

[B32] Hansen A. S. Beery T. Fredman P. Wolf-Watz D. (2022). Outdoor recreation in Sweden during and after the Covid-19 pandemic – management and policy implications. *J. Environ. Plan. Manag.* 65 1–22. 10.1080/09640568.2022.2029736

[B33] Himes A. Muraca B. (2018). Relational values: The key to pluralistic valuation of ecosystem services. *Curr. Opin. Environ. Sustain.* 35 1–7. 10.1016/j.cosust.2018.09.005

[B34] Jarský V. Palátová P. Riedl M. Zahradník D. Rinn R. Hochmalová M. (2022). Forest attendance in the times of COVID-19—a case study on the example of the czech republic. *Int. J. Environ. Res. Public Health* 19:2529. 10.3390/ijerph19052529 35270222 PMC8909629

[B35] Jo J.-H. Lee C.-B. Cho H.-J. Lee J. (2021). Estimation of citizens’ willingness to pay for the implementation of payment for local forest ecosystem services: The case of taxes and donations. *Sustainability* 13:6186. 10.3390/su13116186

[B36] Kabaya K. Hashimoto S. Fukuyo N. Uetake T. Takeuchi K. (2019). Investigating future ecosystem services through participatory scenario building and spatial ecological–economic modelling. *Sustain. Sci.* 14 77–88. 10.1007/s11625-018-0590-1

[B37] Khalfaoui M. Daly-Hassen H. Stiti B. Jebari S. (2020). Toward decision-making support: Valuation and mapping of new management scenarios for Tunisian Cork oak forests. *Forests* 11:197. 10.3390/f11020197

[B38] Kim I. Kwon H. (2021). Assessing the impacts of Urban Land use changes on regional ecosystem services according to Urban Green space policies via the patch-based cellular automata model. *Environ. Manag.* 67 192–204. 10.1007/s00267-020-01394-2 33249532

[B39] Kim J. Son Y. (2021). Assessing and mapping cultural ecosystem services of an urban forest based on narratives from blog posts. *Ecol. Indic.* 129:107983. 10.1016/j.ecolind.2021.107983

[B40] Koprowicz A. Korzeniewicz R. Pusz W. Baranowska M. (2022). Sociodemographic determinants of poles’ attitudes towards the forest during the COVID-19 pandemic. *Int. J. Environ. Res. Public Health* 19:1537. 10.3390/ijerph19031537 35162559 PMC8834990

[B41] Kotera Y. Richardson M. Sheffield D. (2022). Effects of shinrin-yoku (Forest Bathing) and nature therapy on mental health: A systematic review and meta-analysis. *Int. J. Ment. Health Addict.* 20 337–361. 10.1007/s11469-020-00363-4

[B42] Kramer K. Bouriaud L. Feindt P. H. van Wassenaer L. Glanemann N. Hanewinkel M. (2022). Roadmap to develop a stress test for forest ecosystem services supply. *One Earth* 5 25–34. 10.1016/j.oneear.2021.12.009

[B43] Lhoest S. Dufrêne M. Vermeulen C. Oszwald J. Doucet J.-L. Fayolle A. (2019). Perceptions of ecosystem services provided by tropical forests to local populations in Cameroon. *Ecosyst. Serv.* 38:100956. 10.1016/j.ecoser.2019.100956

[B44] Lin J.-C. Chiou C.-R. Chan W.-H. Wu M.-S. (2021). Public perception of forest ecosystem services in Taiwan. *J. Forest Res.* 26 344–350. 10.1080/13416979.2021.1911023

[B45] Lingua E. Bettella F. Pividori M. Marzano R. Garbarino M. Piras M. (2020). “The protective role of forests to reduce rockfall risks and impacts in the alps under a climate change perspective,” in *Climate change, hazards and adaptation options: Handling the impacts of a changing climate climate change management*, eds Leal Filho W. Nagy G. J. Borga M. Chávez Muñoz P. D. Magnuszewski A. (Cham: Springer International Publishing), 333–347. 10.1007/978-3-030-37425-9_18

[B46] Liu C. Li J. Steele W. Fang X. (2018). A study on Chinese consumer preferences for food traceability information using best-worst scaling. *PLoS One* 13:e0206793. 10.1371/journal.pone.0206793 30388166 PMC6214548

[B47] Liu W.-Y. Yu H.-W. Hsieh C.-M. (2021). Evaluating forest visitors’ place attachment, recreational activities, and travel intentions under different climate scenarios. *Forests* 12:171. 10.3390/f12020171

[B48] Louviere J. J. Flynn T. N. Marley A. A. J. (2015). *Best-worst scaling: Theory, methods and applications.* Cambridge, MA: Cambridge University Press. 10.1017/CBO9781107337855

[B49] Magnenat L. (2021). “Think like a mountain“-” to think of Oedipus”: A psychoanalytic contribution to environmental ethics. *Int. J. Psychoanal.* 102 734–754. 10.1080/00207578.2021.1924064 34357844

[B50] Malandra F. Vitali A. Urbinati C. Weisberg P. J. Garbarino M. (2019). Patterns and drivers of forest landscape change in the Apennines range, Italy. *Reg. Environ. Change* 19 1973–1985. 10.1007/s10113-019-01531-6

[B51] Marín A. I. Abdul Malak D. Bastrup-Birk A. Chirici G. Barbati A. Kleeschulte S. (2021). Mapping forest condition in Europe: Methodological developments in support to forest biodiversity assessments. *Ecol. Indic.* 128:107839. 10.1016/j.ecolind.2021.107839

[B52] Marley A. Louviere J. (2005). Some probabilistic models of best, worst, and best-worst choices. *J. Math. Psychol.* 49 464–480. 10.1016/j.jmp.2005.05.003

[B53] Massaglia S. Borra D. Peano C. Sottile F. Merlino V. M. (2019). Consumer preference heterogeneity evaluation in fruit and vegetable purchasing decisions using the best–worst approach. *Foods* 8:266. 10.3390/foods8070266 31323883 PMC6678484

[B54] Millennium Ecosystem Assessment (MEA) (2005). *Ecosystems and human well-being.* Washington DC: Island press.

[B55] Mori T. Tsuge T. (2017). Best–worst scaling survey of preferences regarding the adverse effects of tobacco use in China. *SSM Popul. Health* 3 624–632. 10.1016/j.ssmph.2017.07.011 29349250 PMC5769045

[B56] Müller A. Olschewski R. Unterberger C. Knoke T. (2020). The valuation of forest ecosystem services as a tool for management planning – A choice experiment. *J. Environ. Manag.* 271:111008. 10.1016/j.jenvman.2020.111008 32778292

[B57] Muro A. Feliu-Soler A. Canals J. Parrado E. Sanz A. (2022). Psychological benefits of Forest bathing during the COVID-19 pandemic: A pilot study in a mediterranean forest close to urban areas. *J. Forest Res.* 27 71–75. 10.1080/13416979.2021.1996516

[B58] Nummelin T. Hänninen R. Kniivilä M. (2021). Exploring forest sector research subjects and trends from 2000 to 2019 using topic modeling. *Curr. Forestry Rep.* 7 267–281. 10.1007/s40725-021-00152-9

[B59] Orme B. K. (2009). *MaxDiff analysis: Simple counting, individual-level logit, and HB.* Sequim, WA: Sawtooth Software.

[B60] Pastorella F. Giacovelli G. Maesano M. Paletto A. Vivona S. Veltri A. (2016). Social perception of forest multifunctionality in southern Italy: The case of Calabria Region. *J. Forest Sci.* 62 366–379.

[B61] Pereira H. M. Rosa I. M. D. Martins I. S. Kim H. Leadley P. Popp A. (2020). Global trends in biodiversity and ecosystem services from 1900 to 2050. *bioRxiv* [Preprint] 10.1101/2020.04.14.031716 38662818

[B62] Piaggio M. Siikamäki J. (2021). The value of forest water purification ecosystem services in Costa Rica. *Sci. Total Environ.* 789:147952. 10.1016/j.scitotenv.2021.147952 34058576

[B63] Prakoso S. G. Timorria I. F. Murtyantoro A. P. (2021). Social media interconnection between people: Greta Thunberg’s influence on the climate movement. *IOP Conf. Ser. Earth Environ. Sci.* 905:012136. 10.1088/1755-1315/905/1/012136

[B64] Rijal S. Rimal B. Acharya R. P. Stork N. E. (2021). Land use/land cover change and ecosystem services in the Bagmati River Basin, Nepal. *Environ. Monit. Assess.* 193:651. 10.1007/s10661-021-09441-z 34523026

[B65] Rimal B. Keshtkar H. Stork N. Rijal S. (2021). Forest cover and sustainable development in the lumbini province, Nepal: Past, present and future. *Remote Sens.* 13:4093. 10.3390/rs13204093

[B66] Roviello V. Roviello G. N. (2022). Less COVID-19 deaths in southern and insular Italy explained by forest bathing, Mediterranean environment, and antiviral plant volatile organic compounds. *Environ. Chem. Lett.* 20 7–17. 10.1007/s10311-021-01309-5 34483793 PMC8408569

[B67] Sabherwal A. Ballew M. T. van der Linden S. Gustafson A. Goldberg M. H. Maibach E. W. (2021). The greta thunberg effect: Familiarity with Greta Thunberg predicts intentions to engage in climate activism in the United States. *J. Appl. Soc. Psychol.* 51 321–333. 10.1111/jasp.12737

[B68] Sacchelli S. Borghi C. Grilli G. (2021). Prevention of erosion in mountain basins: A spatial-based tool to support payments for forest ecosystem services. *J. For. Sci.* 67 258–271. 10.17221/5/2021-JFS

[B69] Santos Vieira F. A. Vinhas Santos D. T. Bragagnolo C. Campos-Silva J. V. Henriques Correia R. A. Jepson P. (2021). Social media data reveals multiple cultural services along the 8.500 kilometers of Brazilian coastline. *Ocean. Coast. Manag.* 214:105918. 10.1016/j.ocecoaman.2021.105918

[B70] Scheidl C. Heiser M. Vospernik S. Lauss E. Perzl F. Kofler A. (2020). Assessing the protective role of alpine forests against rockfall at regional scale. *Eur. J. Forest Res.* 139 969–980. 10.1007/s10342-020-01299-z

[B71] Schirpke U. Tscholl S. Tasser E. (2020). Spatio-temporal changes in ecosystem service values: Effects of land-use changes from past to future (1860–2100). *J. Environ. Manag.* 272:111068. 10.1016/j.jenvman.2020.111068 32854880

[B72] Sharafatmandrad M. Khosravi Mashizi A. (2021). Ecological succession regulates the relationship between biodiversity and supporting services in arid ecosystems. *Arab. J. Geosci.* 14:1370. 10.1007/s12517-021-07796-8

[B73] Shuibul Qarnain S. Muthuvel S. Bathrinath S. (2021). Modelling of driving factors for energy efficiency in buildings using Best Worst Method. *Mater. Today Proc.* 39 137–141. 10.1016/j.matpr.2020.06.400

[B74] Soto J. R. Escobedo F. J. Khachatryan H. Adams D. C. (2018). Consumer demand for urban forest ecosystem services and disservices: Examining trade-offs using choice experiments and best-worst scaling. *Ecosyst. Serv.* 29 31–39. 10.1016/j.ecoser.2017.11.009

[B75] Tabacco E. Merlino V. M. Coppa M. Massaglia S. Borreani G. (2021). Analyses of consumers’ preferences and of the correspondence between direct and indirect label claims and the fatty acid profile of milk in large retail chains in northern Italy. *J. Dairy Sci.* 104 12216–12235. 10.3168/jds.2021-20191 34593234

[B76] Tattoni C. Grilli G. Araña J. Ciolli M. (2021). The landscape change in the alps—what postcards have to say about aesthetic preference. *Sustainability* 13:7426. 10.3390/su13137426

[B77] Tauro A. Gómez-Baggethun E. García-Frapolli E. Chavero E. L. Balvanera P. (2018). Unraveling heterogeneity in the importance of ecosystem services: Individual views of smallholders. *Ecol. Soc.* 23:11.

[B78] The Economics of Ecosystems and Biodiversity (TEEB). (2010). *The economics of ecosystems and biodiversity ecological and economic foundations.* London: Earthscan.

[B79] Umberger W. J. Stringer R. Mueller S. C. (eds). (2010). *Using best-worst scaling to determine market channel choice by small farmers in Indonesia*. St. Paul, MN: AgEcon Search. 10.22004/ag.econ.90853

[B80] VanderWilde C. P. Newell J. P. (2021). Ecosystem services and life cycle assessment: A bibliometric review. *Resourc. Conserv. Recycl.* 169:105461. 10.1016/j.resconrec.2021.105461

[B81] Vos S. Bijnens E. M. Renaers E. Croons H. Van Der Stukken C. Martens D. S. (2022). Residential green space is associated with a buffering effect on stress responses during the COVID-19 pandemic in mothers of young children, a prospective study. *Environ. Res.* 208:112603. 10.1016/j.envres.2021.112603 34995548 PMC8730780

[B82] Yang S. Zhao W. Pereira P. Liu Y. (2019). Socio-cultural valuation of rural and urban perception on ecosystem services and human well-being in Yanhe watershed of China. *J. Environ. Manag.* 251:109615. 10.1016/j.jenvman.2019.109615 31581043

[B83] Yoshimura M. Matsuura T. Sugimura K. (2021). Attitudes to forest conditions and fishing activities in the mountain area in Japan. *Fish. Res.* 244:106125. 10.1016/j.fishres.2021.106125

[B84] Zhang H. Pang Q. Long H. Zhu H. Gao X. Li X. (2019). Local residents’ perceptions for ecosystem services: A case study of fenghe river watershed. *Int. J. Environ. Res. Public Health* 16:3602. 10.3390/ijerph16193602 31561464 PMC6801443

