# Corrigendum: Civil society's perception of forest ecosystem services. A case study in the Western Alps

**DOI:** 10.3389/fpsyg.2022.1107019

**Published:** 2022-12-20

**Authors:** Stefano Bruzzese, Simone Blanc, Valentina Maria Merlino, Stefano Massaglia, Filippo Brun

**Affiliations:** Department of Agricultural, Forest and Food Sciences (DISAFA), University of Turin, Turin, Italy

**Keywords:** forest ecosystem services, Best-Worst Scaling, latent class analysis, civil society, awareness, perception

In the published article, an author name was incorrectly written as “Valentino Maria Merlino.” The correct spelling is “Valentina Maria Merlino.”

The authors apologize for this error and state that this does not change the scientific conclusions of the article in any way. The original article has been updated.

