# Supplementary material for: Civil society’s perception of forest ecosystem services. A case study in the Western Alps
Source: Front Psychol. 2022 Sep 29;13:1000043. doi: 10.3389/fpsyg.2022.1000043 (PMC9586207; doi:10.3389/fpsyg.2022.1000043)
Supplement: Supplementary file 1 [file Data_Sheet_1.PDF]

APPENDIX A

Example of a questionnaire version employed in the data collection (translated).

A) Which of the following services provided by the mountain-forest context/ecosystem do you consider most important?

Tick the ONE reason that influences your choice the MOST and the ONE reason that influences your choice the LEAST:

MOST IMPORTANT (one answer)	CHARACTERISTIC	LEAST IMPORTANT (one answer)
○	Drinking water at the home tap	○
○	Psychophysical health (e.g. reduces stress and strengthens the immune system)	○
○	Climate change mitigation (e.g. carbon storage)	○
○	Recreational tourism (e.g. hiking, mountain biking and camping)	○

Tick the ONE reason that influences your choice the MOST and the ONE reason that influences your choice the LEAST:

MOST IMPORTANT (one answer)	CHARACTERISTIC	LEAST IMPORTANT (one answer)
○	Psychophysical health (e.g. reduces stress and strengthens the immune system)	○
○	Aesthetic quality of landscape/beauty	○
○	Fuel (e.g. firewood, pellets and wood chips)	○
○	Protection against natural hazards (e.g. avalanches, rockfalls and landslides)	○

Tick the ONE reason that influences your choice the MOST and the ONE reason that influences your choice the LEAST:

PIU' INFLUENTE (una risposta)	CARATTERISTICA	MENO INFLUENTE (una risposta)
○	Biodiversity (e.g. plant and animal habitats)	○
○	Fuels (e.g. firewood, pellets and wood chips)	○
○	Psychophysical health (e.g. reduces stress and strengthens the immune system)	○
○	Food (e.g. mushrooms, small fruits, fish and game)	○

Tick the ONE reason that influences your choice the MOST and the ONE reason that influences your choice the LEAST:

MOST IMPORTANT (one answer)	CHARACTERISTIC	LEAST IMPORTANT (one answer)
○	Climate change mitigation (carbon storage)	○
○	Drinking water at the home tap	○
○	Aesthetic quality of landscape/beauty	○
○	Raw materials (e.g. construction timber, carpentry and handicrafts)	○

Tick the ONE reason that influences your choice the MOST and the ONE reason that influences your choice the LEAST:

MOST IMPORTANT (one answer)	CHARACTERISTIC	LEAST IMPORTANT (one answer)
○	Food (e.g. mushrooms, small fruits, fish and game)	○
○	Climate change mitigation (e.g. carbon storage)	○
○	Reduction of calamitous events (e.g. floods)	○
○	Spiritual and religious (e.g. pilgrimage and religious retreats)	○

Tick the ONE reason that influences your choice the MOST and the ONE reason that influences your choice the LEAST:

MOST IMPORTANT (one answer)	CHARACTERISTIC	LEAST IMPORTANT (one answer)
○	Aesthetic quality of landscape/beauty	○
○	Fuel (e.g. firewood, pellets and wood chips)	○
○	Biodiversity (e.g. plant and animal habitats)	○
○	Reduction of calamitous events (e.g. floods)	○

Tick the ONE reason that influences your choice the MOST and the ONE reason that influences your choice the LEAST:

MOST IMPORTANT (one answer)	CHARACTERISTIC	LEAST IMPORTANT (one answer)
○	Raw materials (e.g. construction timber, carpentry and objects)	○
○	Natural hazard protection (e.g. avalanches, rockfalls and landslides)	○
○	Food (e.g. mushrooms, small fruits, fish and game)	○
○	Spiritual and religious (e.g. pilgrimage and religious retreats)	○

Tick the ONE reason that influences your choice the MOST and the ONE reason that influences your choice the LEAST:

MOST IMPORTANT (one answer)	CHARACTERISTIC	LEAST IMPORTANT (one answer)
○	Biodiversity (e.g. plant and animal habitats)	○
○	Protection against natural hazards (e.g. avalanches, rockfalls and landslides)	○
○	Drinking water at the home tap	○
○	Recreational tourism (e.g. hiking, mountain biking and camping)	○

Tick the ONE reason that influences your choice the MOST and the ONE reason that influences your choice the LEAST:

MOST IMPORTANT (one answer)	CHARACTERISTIC	LEAST IMPORTANT (one answer)
○	Disaster mitigation (e.g. flooding)	○
○	Raw materials (e.g. building timber, carpentry and handicrafts)	○
○	Spiritual and religious (e.g. pilgrimage and religious retreats)	○
○	Tourist-recreational (e.g. hiking, mountain biking and camping)	○

B) SOCIO-DEMOGRAPHIC DATA

B1) Age _____________ B2) Sex M ☐ F ☐

B3) How many people are in your family? _____________

B4) Postcode or city of residence _____________

B5) What is your educational level?

☐	Elementary school certificate		Degree (BSc, MSc)
☐	High school certificate	☐	Postgraduate degree (Master, Doctorate, Specialisation)

B6) Job description ____

B7) Which of the following net monthly household income brackets do you fall into?

☐	Less than 1,000 €	☐	From 4,001 to 6,000 €
☐	From 1,001 to 2,000 €	☐	More than 6,000 €
☐	From 2,001 to 3,000 €	☐	No answer
☐	From 3,001 to 4,000 €		

B8) How often do you visit the Valley in a year? 1 ☐ 2-5 ☐ 6-10 ☐ 11-20 ☐ more than 20 ☐
